# Characterising skeletal muscle haemoglobin saturation during exercise using near-infrared spectroscopy in chronic kidney disease

**DOI:** 10.1007/s10157-018-1612-0

**Published:** 2018-06-30

**Authors:** Thomas J. Wilkinson, Alice E. M. White, Daniel G. D. Nixon, Douglas W. Gould, Emma L. Watson, Alice C. Smith

**Affiliations:** 1 Leicester Kidney Lifestyle Team, Department of Infection, Immunity, and Inflammation, University of Leicester, Leicester, LE5 4PW UK; 2 John Walls Renal Unit, University Hospitals of Leicester, Leicester General Hospital, Leicester, UK

**Keywords:** Near-infrared spectroscopy, Oxygen saturation, Haemoglobin, Exercise, Chronic kidney disease

## Abstract

**Background:**

Chronic kidney disease (CKD) patients have reduced exercise capacity. Possible contributing factors may include impaired muscle O_2_ utilisation through reduced mitochondria number and/or function slowing the restoration of muscle ATP concentrations via oxidative phosphorylation. Using near-infrared spectroscopy (NIRS), we explored changes in skeletal muscle haemoglobin/myoglobin O_2_ saturation (SMO_2_%) during exercise.

**Methods:**

24 CKD patients [58.3 (± 16.5) years, eGFR 56.4 (± 22.3) ml/min/1.73 m^2^] completed the incremental shuttle walk test (ISWT) as a marker of exercise capacity. Using NIRS, SMO_2_% was measured continuously before, during, and after (recovery) exercise. Exploratory differences were investigated between exercise capacity tertiles in CKD, and compared with six healthy controls.

**Results:**

We identified two discrete phases; a decline in SMO_2_% during incremental exercise, followed by rapid increase upon cessation (recovery). Compared to patients with low exercise capacity [distance walked during ISWT, 269.0 (± 35.9) m], patients with a higher exercise capacity [727.1 (± 38.1) m] took 45% longer to reach their minimum SMO_2_% (*P* = .038) and recovered (half-time recovery) 79% faster (*P* = .046). Compared to controls, CKD patients took significantly 56% longer to recover (i.e., restore SMO_2_% to baseline, full recovery) (*P* = .014).

**Conclusions:**

Using NIRS, we have determined for the first time in CKD, that favourable SMO_2_% kinetics (slower deoxygenation rate, quicker recovery) are associated with greater exercise capacity. These dysfunctional kinetics may indicate reduced mitochondria capacity to perform oxidative phosphorylation—a process essential for carrying out even simple activities of daily living. Accordingly, NIRS may provide a simple, low cost, and non-invasive means to evaluate muscle O_2_ kinetics in CKD.

## Introduction

Chronic kidney disease (CKD) is associated with premature mortality, multi-morbidity, and reduced quality of life [1, 2]. Patients with CKD are characteristically physically inactive [3] and have reduced physical functioning and exercise capacity [4]. In CKD, poor exercise capacity and performance are independently associated with adverse clinical outcomes and an impaired ability to complete activities of daily living (ADLs) [5–7].

A key determinant of aerobic capacity, particularly of prolonged or progressive duration, is the ability to adequately supply the working skeletal muscles with oxygen (O_2_) to regenerate adenosine triphosphate (ATP), fundamental in muscle contraction [8, 9]. This process largely occurs in the mitochondria respiratory chain [10, 11] via oxidative phosphorylation [12]. Skeletal muscle mitochondrial dysfunction is well established across all stages of CKD [13–18], and alterations in mitochondrial number and function can lead to impairments in oxidative phosphorylation [17, 19]. Mitochondrial dysfunction may also result in increased reactive O_2_ species production, altered cellular redox state, deregulation of calcium homeostasis, triggering of mitoptosis/apoptosis [11, 13, 14], and reductions of muscle function [13, 17].

Therefore, assessing mitochondria dysfunction appears of utmost importance in the progression of CKD-related changes in muscle function, and requires the development of clinically relevant, reproducible, and accurate assessments of mitochondria capacity [9, 12]. Skeletal muscle mitochondria have historically been evaluated using at the tissue level [9], however, non-invasive tools such as 31P-magnetic resonance spectroscopy (31P^MRS^) allow for in vivo measurement. However, the use of this methodology is severely restricted by its cost and accessibility [12].

Near-infrared spectroscopy (NIRS) can be used to measure muscle-oxidative metabolism in vivo [9, 11, 12, 20]. NIRS measures the attenuation (reduction in intensity) of light in the near-infrared spectrum to quantify the chromophores, mainly haemoglobin (Hb) and myoglobin (Mb), present in the muscle tissue [21]. The absorbance of near-infrared light differs depending on whether the molecules are in an oxygenated or deoxygenated state. Unfortunately, the spectral absorbance of Hb and Mb is indistinguishable and attenuation in the muscle is attributed to both [20]. Recent data suggests Mb contributes ~ 50–70% of the NIRS signal and is likely to increase during exercise [21]. As such, NIRS can be considered to reflect local tissue oxygenation inclusive of Hb and Mb [22]. By measuring skeletal muscle O_2_ saturation (SMO_2_%) (i.e., the % of oxygenated Hb/Mb) during exercise, NIRS allows the non-invasive exploration of the balance between O_2_ delivery and demand [9, 23, 24].

There is an increasing clinical interest in the ability of NIRS [25] to quickly and non-invasively measure skeletal muscle mitochondrial function in clinical populations that may be affected by decrements in oxidative capacity [9, 12, 25–29]. NIRS-derived estimates of mitochondrial oxidative capacity are based on the relationship between oxidative phosphorylation and phosphocreatine (PCr) recovery following exercise [12]. Recent studies have demonstrated the post-exercise recovery kinetics of oxygen metabolism by NIRS is a valid and reproducible proxy model of assessing in vivo mitochondrial respiratory capacity and skeletal muscle-oxidative phosphorylation [9, 11, 20, 30]. Strong agreeability between NIRS and PCr measured by 31P^MRS^ have been observed [9, 11, 20, 31].

Delayed skeletal muscle oxygen metabolism recovery following exercise has been observed in different clinical populations, such as peripheral artery disease (PAD) and chronic obstructive pulmonary disease (COPD) [23, 24, 32, 33]. As many daily tasks are characterized by repetitive activities (e.g., stair climbing), this delayed recovery is likely to be an important contributor to poor exercise tolerance and reduced ability to perform ADLs in CKD. Along with delayed SMO_2_% recovery, the rate of SMO_2_% decline during exercise is also a key parameter in muscle O_2_ metabolism [23, 24]. During progressive exercise, a steady decline in SMO_2_% represents an imbalance between supply and utilization [34]. The minimum SMO_2_% reached occurs at/near maximum exercise capacity [35–37], and the observed time to reach the minimum SMO_2_% has been shown to be more pronounced in PAD [24], chronic heart failure (CHF) [36], and COPD [37] patients compared to controls. A more rapid and distinct SMO_2_% decline may indicate imbalances between muscle O_2_ supply and utilization, and early onset of anaerobic metabolism [35, 36].

No research to date has investigated NIRS assessed SMO_2_% changes during exercise in a CKD population. Given the high prevalence of skeletal muscle dysfunction and reduced exercise capacity in CKD, research into possible non-invasive markers and mechanisms is warranted. As such, the purpose of this exploratory trial was to (1) describe calf SMO_2_% changes during incremental exercise; (2) determine the association of these SMO_2_% changes with exercise capacity and clinical parameters; and (3) preliminary explore any differences between CKD and a small group of healthy controls, and differences in CKD patients with diabetes mellitus type II (T2DM).

## Materials and methods

### Population

Non-dialysis-dependent CKD patients attending nephrology outpatient clinics based at the Leicester General Hospital, UK were approached to take part. Exclusion criteria included: < 18 years old, pregnancy, kidney transplant within 6 months, visual or hearing impairment, and inability to give informed consent. Healthy controls (HC) (no prior diagnosis of kidney disease) were sought via local adverts and community groups. This is an exploratory sub-study of a trial investigating immune response to exercise in renal patients [38] (ISRCTN38935454). The study was approved by a National Research Ethics Committee (15/EM/0391) and conducted in accordance with the Declaration of Helsinki.

### Exercise protocol

Patients underwent baseline assessments of anthropometry and completed the Incremental Shuttle Walk Test (ISWT) [39]. During the ISWT, the patient walked a distance of 10 m back and forth and around two cones. The walking pace was governed by an external audio tone and increased by a rate of 0.17 m/s every minute for 12 stages or until the patient could no longer keep up with the pace due to volitional exhaustion, pain, or other exertion-limiting symptoms. Upon cessation of the test, patients were immediately seated.

The ISWT is a recognised proxy measure of aerobic capacity, and has been extensively validated against cardiopulmonary exercise testing in numerous clinical populations (e.g., [39, 40]). To assess the relationship between aerobic capacity and NIRS-derived parameters (described below), patients were divided into tertiles (‘upper’, ‘middle’, and ‘lower’).

### Outcome measures

#### Skeletal muscle O_2_ saturation kinetics

Calf muscle SMO_2_% was measured before, during, and after the ISWT using a wireless continuous-wave NIRS spectrometer (BSXInsight, USA). This specific device has been validated in healthy participants [41]. The calf muscle was selected as it plays a key role in walking ability and gait [42] and is less influenced by adiposity when compared to other parts of the body [20].

**Fig. 1:**
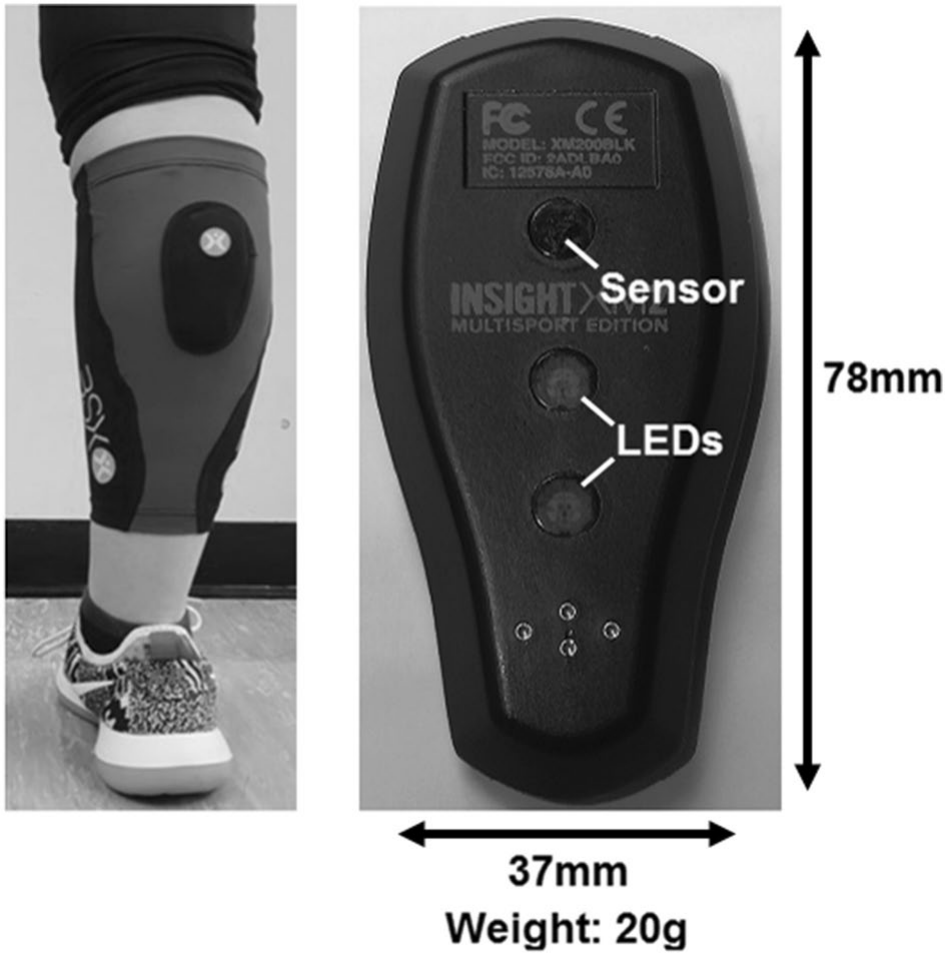
Relative size and application of BSXInsight NIRS device to lower leg. *LED* light-emitting diode

Positioned on the skin, the NIRS device uses specific, calibrated wavelengths (700–1000 nm) of near-infrared light to penetrate the tissue. The source of this near-infrared light is emitted by two light-emitting diodes (LEDs) on the back of the device (Fig. 1). This light is absorbed by chromophores (primarily Hb). A modified Lambert–Beer law can be used to calculate the chromophore’s absorption and relative concentration.

$$\Delta c~=\frac{{\Delta {\text{OD}}\lambda }}{{\varepsilon \lambda \cdot L \cdot {\text{DPF}}}}$$

where here OD*λ* represents the oxygen-independent optical losses due to scattering and absorption in the tissue; *ελ* the chromophore’s extinction coefficient (in µm^−1^ cm^−1^); *c* is the concentration (in µm) of the chromophore; *L* the distance (in cm) between light entry and exit points; DPF is the differential pathlength factor (DPF); and *λ* is the wavelength used (in nm) [9].

The spectra of the two main chromophores [oxyhemoglobin (O_2_Hb) and deoxyhemoglobin (HHb)] is used quantify the percentage of Hb/Mb O_2_ [i.e., O_2_Hb/(O_2_Hb + HHb) × 100] saturation in the microvasculature [9, 20].

To ensure consistent replacement, the device was fitted at the widest measured point of the gastro-soleus complex in line with the Achilles tendon on the self-reported dominant leg. The device was covered with a black cloth to remove any contamination of ambient light and secured in place using foam medical bandage (Fig. 1).

For the main trial [38] patients attended a second visit 7 days (median) later. We used this opportunity to collect another resting baseline SMO_2_% sample and calculate the test–retest reliability of the device. Baseline coefficient of variation (CV) was 7.6%. The intraclass correlation coefficient (mean rating, absolute agreement, two-way mixed effects model) was .377. The minimal detectable difference at a group level was 3.0%.

**Fig. 2:**
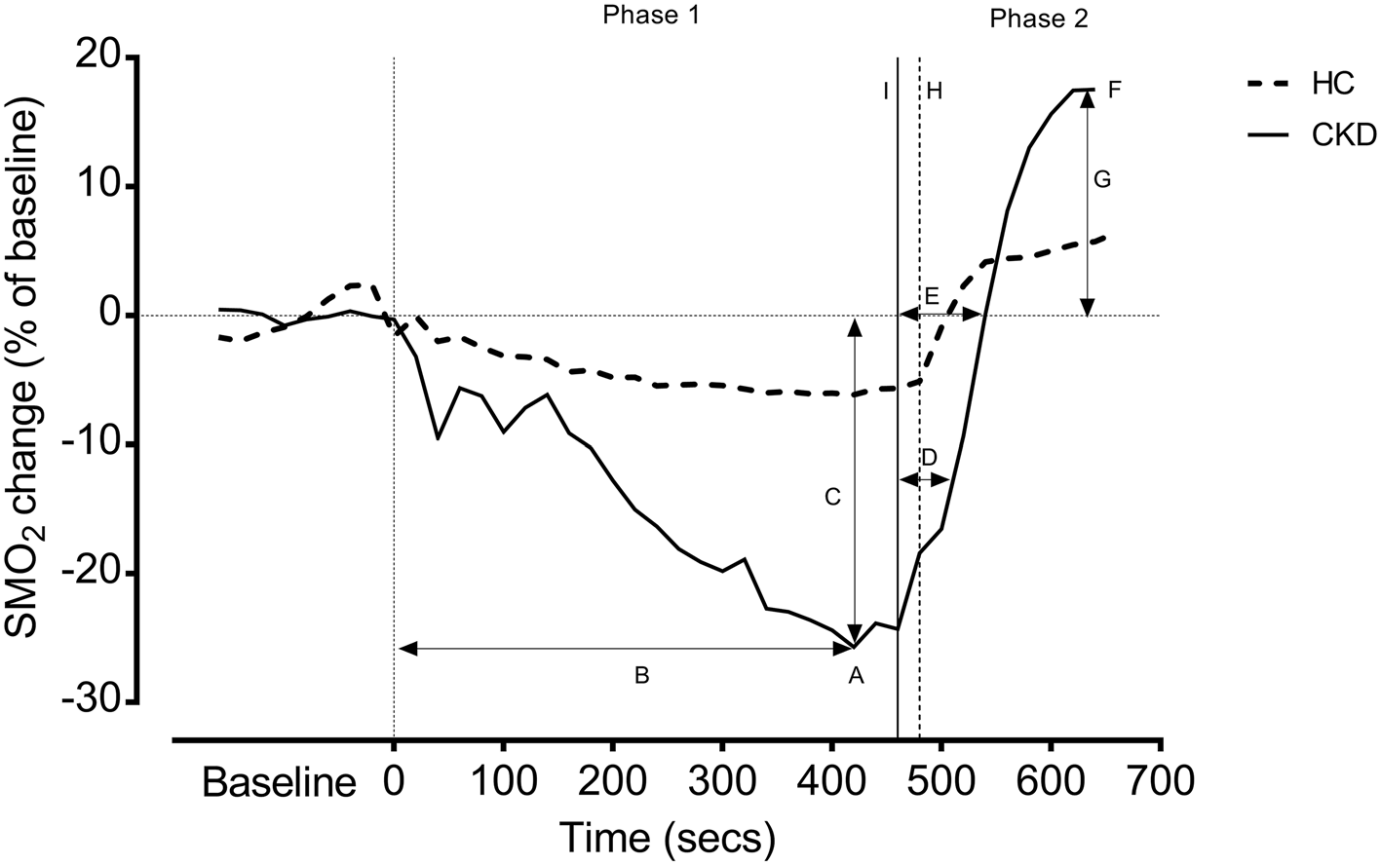
Representative example SMO_2_% for example HC and CKD patient during incremental exercise. Data presented as SMO_2_% change (as a % of baseline); to reduce noise, data is shown as 20 s average. *HC* healthy control, *CKD* chronic kidney disease. Baseline refers to 3 min sitting measure of calf SMO_2_% obtained at rest. Phase 1 refers to the incremental shuttle walk test (ISWT); Phase 2 refers to the 3 min recovery period. *A* = the minimum SMO_2_% value; *B* = time taken to reach the minimum value; *C* = the absolute and percentage drops in SMO_2_% from rest to the minimum exercise value; *D* = recovery ½ time; *E* = recovery full time; *F* = SMO_2_% value reached after 3 min recovery; *G* = % overshoot above baseline; *H* = end of ISWT for HC; *I* = end of ISWT for CKD

SMO_2_% variables, including passive recovery times (taken over 3 min whilst the participant was seated immediately after finishing the ISWT), were obtained based on the previous literature [23, 24] and shown in Fig. 2.

#### Anthropometry

Body mass and height were measured per standard techniques.

#### Clinical parameters

Clinical data were extracted from participant-recorded medical history. These included disease aetiology and comorbidities. A blood sample was collected prior to the ISWT, and used to measure renal function (eGFR) and Hb. In a sub-sample of CKD patients (*n* = 10), haematological iron status was analysed.

### Statistical analysis

As an exploratory sub-study utilising non-dialysis patients from a larger trial [38], an a priori sample size was not calculated. However, a post hoc calculation using the time to reach minimum SMO_2_% between the exercise capacity groups (effect size *d* = 1.4) revealed an achieved power (*β*) of 0.92. Thus, indicating adequate power to detect differences among groups for the time to minimum SMO_2_%. Unless otherwise stated, data are presented as estimated marginal means (± SE) taken from analysis of covariance (ANCOVA). Non-normally distributed data (Shapiro–Wilk test) was transformed as appropriate. Controlling for age and sex disparities, differences between aerobic capacity groups were assessed using ANCOVA. If a between-group difference was detected, Tukey post hoc pairwise comparisons were used. Differences between CKD and HC, and between patients with and without T2DM, were measured by ANCOVA. Due to differences between groups, age and sex were used as covariates. Given the small sample, Cohens *d* was calculated as an effect size to provide a better interpretation of the differences. A ‘large’ effect size was determined as ≥ 0.8, with ‘very large’ defined as ≥ 1.3 [43]. Pearson’s Chi-square tests were used to measure differences in categorical variables. Figure 2 presents SMO_2_% changes (20 s average) relative to a 3-min averaged baseline value for an example CKD patient and HC. Both were matched for approximate distance walked to provide a better interpretation of differences. Data were analysed using SPSS (v24). Statistical significance was set at *P* < .050.

## Results

125 CKD patients were approached, of which 101 patients declined and 24 consented. Six HCs were recruited to provide a small exploratory comparative group. Participant characteristics are shown in Table 1. Overall, HC were ~ 11 years younger (*P* = .498) with a larger frequency of females (*P* = .044). Confirming their status as ‘healthy’, the control group self-reported no incidence of T2DM or hypertension. With the exception of one individual (88 ml/min/1.73 m^2^), all controls had an eGFR of ≥ 90 ml/min/1.73 m^2^. No participants were anaemic (Hb < 120 g/l for female, < 130 g/l for males).

**Table 1:** Participant characteristics

	CKD (*n* = 24)	HC (*n* = 6)	*P*
Age (years)	58.3 (± 16.5)	47.2 (± 19.8)	.498
Sex, *n* female (%)	9 (38%)	5 (83%)	.044*
BMI (kg/m^2^)	29.0 (± 6.3)	25.8 (± 3.7)	.259
Ethnicity			
White British, *n* (%)	20 (84%)	5 (83%)	.518^a^
White European, *n* (%)	2 (8%)	0 (0%)	
Asian, *n* (%)	2 (8%)	1 (17%)	
Disease aetiology			
Granulomatosis with polyangitis, *n* (%)	2 (8%)	–	–
Interstitial nephritis, *n* (%)	2 (8%)	–	–
IgA nephropathy, *n* (%)	13 (54%)	–	–
Polycystic kidney disease, *n* (%)	1 (4%)	–	–
Other, *n* (%)	3 (13%)	–	–
Unknown/aetiology uncertain, *n* (%)	3 (13%)	–	–
Comorbidities			
Diabetes mellitus type II, *n* (%)	7 (29%)	0 (0%)	.131
Hypertension, *n* (%)	12 (50%)	0 (0%)	.025*
Clinical parameters			
eGFR (ml/min/1.73 m^2^)	56.4 (± 22.3)	88.8 (± 2.7)	.004*
Hb (mg/dl)	140.4 (± 14.0)	138.0 (± 8.6)	.725
Ferritin (µg/l)^b^	125.5 (± 165.6)	–	–
Iron (µg/dl)^b^	12.0 (± 3.6)	–	–
Transferrin (g/dl)^b^	2.3 (± 0.3)	–	–
Transferrin saturation ratio (%)^b^	20.9 (± 7.7)	–	–
Blood pressure (systolic) (mmHg)	136.0 (± 18.6)	127.5 (± 19.3)	.329
Blood pressure (diastolic) (mmHg)	79.6 (± 9.3)	77.7 (± 10.2)	.629
Heart rate (bpm)	64.4 (± 10.5)	69.0 (± 9.6)	.337

Unless stated, data presented as mean (± SD)

*CKD* chronic kidney disease, *HC* healthy control, *BMI* body mass index, *eGFR* estimated glomerular filtration rate, *Hb* haemoglobin

*P < .050

^a^Results from Pearson’s Chi-square test

^b^Anaemic parameters only available for *n* = 10 CKD

### Skeletal muscle oxygenation kinetic responses to incremental exercise in CKD

We identified two discrete phases during incremental exercise (Tables 2, 3; Fig. 2):

**Table 2:** Skeletal muscle oxygen saturation characteristics and incremental exercise performance in CKD patients

	Aerobic capacity (based on the ISWT) tertiles			
	‘Lower’ (*n* = 8)	‘Middle’ (*n* = 8)	‘Upper’ (*n* = 8)	*P*
Age (years)	72.0 (± 8.1)	57.5 (± 13.7)^a,*^	45.3 (15.2) ^a,*^	.002*
Sex, *n* female (%)	3 (38%)	4 (50%)	2 (25%)	.587
eGFR (ml/min/kg/1.73 m^2^)	48.8 (± 21.9)	58.0 (± 16.4)	64.0 (± 26.4)	.435
ISWT (distance walked, m)	269.0 (± 35.9)	479.6 (± 36.0)^a,*^	727.1 (± 38.1)^a,*, b,*^	< .001*
Baseline SMO_2_ (%)	66.6 (± 3.0)	70.3 (± 2.4)	73.6 (± 2.8)	.326
Minimum % reached (%)	62.5 (± 3.2)	60.5 (± 2.5)	64.5 (± 2.9)	.586
Time to min (s)	268.6 (± 65.3)	352.6 (± 52.0)	491.7 (± 60.5)^a,*^	.099
% Drop (%)	9.0 (± 2.1)	9.8 (± 1.7)	8.7 (± 2.0)	.888
% Change (% of baseline)	13.5 (± 3.3)	14.2 (± 2.6)	11.8 (± 3.1)	.844
Recovery ½ time (s)	58.6 (± 12.9)	51.1 (± 10.3)	12.2 (± 11.9)^a,*, b,*^	.046*
Recovery full time (s)	126.6 (± 27.0)	88.2 (± 20.0)	41.1 (± 23.0)^a,*^	.121
% after 3 min recovery (%)	65.7 (± 2.8)	74.3 (± 2.3)^a,*^	78.4 (± 2.6)^a,*^	.025*
Overshoot (% of baseline)	− 0.9 (± 2.3)	5.7 (± 1.8)^,*^	6.6 (± 2.1)^a,*^	.071

Controlling for differences age and sex, data presented as estimated marginal means (± SE)

*CKD* chronic kidney disease, *eGFR* estimated glomerular filtration rate, *ISWT* incremental shuttle walk test, *SMO*_*2*_ skeletal muscle oxygen (%)

*P < .050

^
a,*^Significant (*P* < .050) difference from ‘lower’

^
b,*^Significant (*P* < .050) difference from ‘middle’

**Table 3:** Differences in skeletal muscle oxygen saturation characteristics and incremental exercise performance between CKD and HC

	CKD (*n* = 24)	HC (*n* = 6)	Mean difference (95% CI)^†^	*P*	*d*
ISWT (distance walked, m)	495.7 (± 31.3)	575.6 (± 66.4)	79.9 (− 75.3 to 235.1)	.300	0.5 (M)
Baseline SMO_2_ (%)	70.3 (± 1.4)	73.8 (± 3.1)	3.5 (− 3.6 to 10.5)	.318	0.4 (S)
Minimum % reached (%)	62.4 (± 1.4)	66.4 (± 3.1)	4.0 (− 3.0 to 11.1)	.250	0.4 (S)
Time to min (s)	369.7 (± 33.1)	413.5 (± 73.6)	43.8 (− 125.6 to 213.2)	.599	0.1
% Drop (%)	9.0 (± 0.9)	4.0 (± 2.1)	− 5.0 (− 9.9 to − 0.2)	.041*	1.5 (VL)
% Change (% of baseline)	13.0 (± 1.4)	11.0 (± 3.2)	− 2.0 (− 9.3 to 5.3)	.564	0.5 (M)
Recovery ½ time (s)	41.6 (± 6.6)	17.5 (± 14.7)	− 24.1 (− 57.9 to 9.7)	.013*	0.7 (M)
Recovery full time (s)	83.1 (± 13.5)	36.8 (± 33.0)	− 46.3 (− 121.4 to 28.7)	.014*	0.9 (L)
% after 3 min recovery (%)	73.0 (± 1.5)	77.0 (± 3.2)	4.0 (− 3.4 to 11.5)	.276	0.4 (S)
Overshoot (% of baseline)	3.8 (± 1.1)	3.4 (± 2.5)	− 0.5 (− 6.2 to 5.3)	.872	0.2 (S)

Controlling for differences age and sex, data presented as estimated marginal means (± SE)

*CKD* chronic kidney disease, *HC* healthy control, *95% CI* 95% confidence interval, *ISWT* incremental shuttle walk test, *SMO*_*2*_ skeletal muscle oxygen (%)

^†^Based on estimated marginal means (with 95% CI)

*d* = Cohen’s *d* effect size [small (*S*) ≥ 0.2, medium (*M*) ≥ 0.5, large (*L*) ≥ 0.8, very large (VL) ≥ 1.3)

#### Phase 1 (SMO_2_% decline upon initiation of exercise)

The average 3-min baseline SMO_2_% was 70.3 (± 1.4)%. Upon initiation of exercise, SMO_2_% dropped sharply before stabilizing. As the incremental walking exercise progressed, SMO_2_% steadily declined until it reached an ‘inflection point’; in some patients, this was more notable than others.

Following this point, SMO_2_% seemingly decreased at a greater rate until it reached a nadir of 62.4 (± 1.4)%, a relative drop of ~ 13% from baseline. On average, it took patients 369.7 (± 33.1) s (~ 6 min), to reach this minimum value. Shortly after reaching this value, patients stopped the test voluntarily due to fatigue or inability to keep up with the set pace.

#### Phase 2 (recovery upon cessation of exercise)

Immediately following exercise, SMO_2_% rapidly increased. It took 41.6 (± 6.6) s for patients to recover to ½ the baseline value, and 83.1 (± 13.5) s to return back to baseline level. At the end of the designated 3-min recovery, SMO_2_% was 73.0 (± 1.5)%, a relative increase (‘overshoot’) of 3.8 (± 1.1)% from baseline [mean baseline was 70.3 (± 1.4)%].

### Relationship between skeletal muscle oxygenation kinetics and aerobic capacity

Table 2 shows differences in skeletal muscle oxygenation kinetics in the tertiles of aerobic capacity (based on distance walked in the ISWT). No differences were seen in baseline SMO_2_% values (*P* = .326), the absolute (*P* = .888) and relative (*P* = .844) drop, and the minimum SMO_2_% value reached during incremental exercise (*P* = .586). There was a small effect of group on the time taken to reach the minimum (*P* = .099). Post hoc tests revealed that those with higher aerobic capacity took significantly longer (223.1 s, ~ 3 min 45 s) to reach minimum SMO_2_% than those with the lowest aerobic capacity (491.7 versus 268.6 s, *P* = .038).

Following the cessation of incremental exercise, those with higher aerobic capacity recovered significantly faster than those with poor aerobic capacity (*P* = .046). The time taken to recover to ½ of baseline SMO_2_% was 12.2 s in the upper aerobic capacity group, significantly quicker than both the middle (51.1 s, *P* = .023) and lower (58.6 s, *P* = .030) groups. A similar pattern was observed for full-time recovery (*P* = .045). Those in the upper (*P* = .009) and middle (*P* = .027) aerobic capacity groups also had a significantly higher SMO_2_% value 3 minutes post exercise than the lower group. This equated to larger % ‘overshoots’ of the baseline in both groups (*P* = .044 and .033, respectively).

### Relationship between skeletal muscle oxygenation kinetics and clinical parameters

No clinical parameters, including eGFR, Hb, and anaemia-related measures were associated with time taken to reach minimum SMO_2_% or recovery ½ and full time (data not shown).

### Differences between HC and CKD

There were no significant differences in distance walked during the ISWT (*P* = .300) between HC and CKD patients (Table 3). Despite a similar aerobic capacity, CKD patients experienced a greater SMO_2_% drop [9.0 versus 4.0%, *P* = .041, *d* = 1.5 (‘very large’)] during the exercise. However, as a proportion of baseline SMO_2_%, this was non-significant (*P* = .564). CKD patients took significantly longer to recover (i.e., restore SMO_2_%) than HC; recovery ½ time was 24.1 s longer (*P* = .013), whilst recovery full time was 46.3 s longer [*P* = .014, *d* = 0.9 (‘large’)].

### Differences between CKD patients with and without T2DM

CKD patients with T2DM displayed poorer aerobic capacity than those without [*P* = .088, *d* = 1.4 (‘very large’)] (Table 4). Whilst there were no significant differences in baseline SMO_2_% or SMO_2_% changes during exercise, diabetic CKD patients took significantly longer to recover; recovery ½ time was 35.8 s longer (*P* = .030), whilst recovery full time was 69.4 s longer (*P* = .037). On average, patients with T2DM never fully restored SMO_2_% to their baseline level; unlike those without T2DM where a relative ‘overshoot’ of 6.1% was observed.

**Table 4:** Differences in skeletal muscle oxygen saturation characteristics between CKD patients with and without diabetes mellitus type II

	Diabetic (*n* = 7)	Non-diabetic (*n* = 17)	Mean difference (95% CI)^†^	*P*	*d*
ISWT (distance walked, m)	391.6 (± 62.5)	529.4 (± 37.9)	− 137.8 (− 298.3 to 22.7)	.088	1.4 (VL)
Baseline SMO_2_ (%)	67.8 (± 3.0)	71.5 (± 1.9)	− 3.6 (− 11.6 to 4.4)	.357	0.4 (S)
Minimum % reached (%)	62.3 (± 3.2)	62.6 (± 2.0)	− 0.3 (− 8.7 to 8.1)	.942	0.1
Time to min (s)	284.6 (± 61.4)	415.2 (± 37.6)	− 130.5 (− 292.9 to 31.9)	.109	0.8 (L)
% Drop (%)	10.1 (± 2.1)	8.8 (± 1.3)	1.4 (− 4.1 to 6.9)	.603	0.3 (S)
% Change (% of baseline)	14.8 (± 3.2)	12.4 (± 2.0)	2.4 (− 6.2 to 10.9)	.568	0.3 (S)
Recovery ½ time (s)	64.8 (± 12.1)	29.0 (± 7.4)	35.8 (− 3.9 to 67.7)	.030*	0.8 (L)
Recovery full time (s)	128.9 (± 23.9)	59.5 (± 15.1)	69.4 (− 4.8 to 134.0)	.037*	1.0 (L)
% After 3 min recovery (%)	67.1 (± 2.9)	75.7 (± 1.8)	− 8.6 (− 16.4 to − 0.8)	0.032*	1.0 (L)
Overshoot (% of baseline)	− 0.8 (± 2.2)	6.1 (± 1.3)	− 7.0 (− 12.7 to − 1.3)	0.019*	1.0 (L)

Controlling for differences age and sex, data presented as estimated marginal means (± SE)

*CKD* chronic kidney disease, *95% CI* 95% confidence interval, *ISWT* incremental shuttle walk test, *SMO*_*2*_ skeletal muscle oxygen (%)

^†^Based on estimated marginal means (with 95% CI)

*d* = Cohen’s *d* effect size [small (S) ≥ 0.2, medium (*M*) ≥ 0.5, large (*L*) ≥ 0.8, very large (VL) ≥ 1.3]

## Discussion

For the first time in a CKD population, we have described NIRS-derived skeletal muscle O_2_ saturation changes during and following exercise. We found poor exercise capacity is associated with a quicker and more pronounced deoxygenation time of SMO_2_% during incremental exercise and a slower recovery time following exercise termination. We also identified recovery of muscle O_2_ saturation in CKD was substantially slower than HC. These variables could indicate dysfunctional skeletal muscle Hb/Mb O_2_ saturation kinetics and, given the recognised association with oxidative phosphorylation and PCr resynthesis, may denote reduced mitochondria capacity in CKD.

The mean resting baseline SMO_2_% value in our sample was 70.3%. Using occlusion-derived hypoxia, the absolute human ranges of SMO_2_% are reportedly ~ 3 (minimum) to 84% (maximum) [44]. Whilst there is limited normative data, our CKD value did not differ significantly from that of controls (73.8%). Indeed, previous estimates of resting SMO_2_% range from 62.3 to 74.4% in healthy participants, although slightly lower in other clinical groups (e.g., T2DM, 46.1–50.3% [45]; PAD, 51.0–59.0% [23, 24]). Nevertheless, due to differences in protocols and devices, it is difficult to make reliable comparisons between these values.

Exercise capacity, and the ability to maintain physical performance is governed by the ability to effectively supply the working skeletal muscles with O_2_ for oxidative phosphorylation [8, 9]. As such, the working muscles require an adequate supply so that O_2_ exceeds (or at least matches) demand. As expected, during incremental exercise, we observed a steady decline in SMO_2_% representing an imbalance between supply and utilization [34]. Pertinently, once patients reached a nadir in SMO_2_%, cessation of the test quickly followed due to fatigue and/or inability to keep pace with the test. This minimum value reportedly occurs as maximal aerobic capacity (i.e., VO_2_ peak/max) is neared or reached [35–37]. This response has been observed in other studies (e.g., [34–37, 46–48]).

Previous research [23, 24] has identified that the time taken to reach the minimum SMO_2_% value as a key variable in muscle O_2_ saturation kinetics during exercise. Both COPD [37] and CHF [36] patients had quicker SMO_2_% deoxygenation rates than controls, whilst in PAD patients, quicker deoxygenation was associated with reduced physical performance during a walking task, as well as earlier onset of pain [23, 24]. In agreement, we found CKD patients with higher aerobic capacity took longer to reach their minimum SMO_2_%, possibly indicating an improved O_2_ perfusion, and/or more efficient O_2_ utilization by the working muscles.

Skeletal muscle deoxygenation may also indicate accumulation of blood lactate. It is well established that exercise tolerance is limited by lactate accumulation, and lactate threshold is often cited as an important marker of cardiorespiratory performance [41]. Grassi et al. [47] reported the onset of SMO_2_% deoxygenation correlated with the onset of blood lactate accumulation during incremental exercise in healthy trained males. Similar findings have been observed in CHF patients [36], with lactate accumulation visible as ‘inflection points’. Once lactate threshold is reached, an accelerated SMO_2_% desaturation has been noted, assumed to be associated with lactic acid facilitated O_2_ unloading from the capillary Hb [9, 35, 36]. In our patients, an ‘inflection’ point could be occasionally identified. However, given the relatively untrained status of the sample, lactate accumulation could begin almost immediately upon exercise initiation, and therefore, early SMO_2_% decline may mask obvious ‘inflection points’. Early and rapid acceleration in muscle deoxygenation, as seen in our patients with lower exercise capacity, could indicate premature onset of anaerobic metabolism [35]. Given that the kidney is responsible for some proportion of lactate metabolism, it is likely that inadequate lactate resorption and clearance in impaired kidneys (specifically the proximal tubular) contributes to this rapid accumulation [49].

Another key variable in SMO_2_% kinetics is the recovery time following exercise [23, 24]. We observed that patients with lower aerobic capacity had longer recovery (or reoxygenation) times than those with higher exercise capacity. We also observed that the recovery time of CKD patients was significantly longer than that of HC. This supports previous research that has shown delayed skeletal muscle reoxygenation following exercise in different clinical populations [23, 24, 32, 33]. Slower reoxygenation time is reportedly related to slower kinetics of PCr, as determined by 31P^MRS^ [8, 12, 20, 30, 32, 46]. PCr rate is a function of mitochondrial ATP production [9], and therefore, slower reoxygenation kinetics during exercise recovery has been considered an index of reduced skeletal muscle oxidative performance [8, 9, 12, 20, 30, 34]. Dysfunction of mitochondria has been previously reported in CKD [15–18], and therefore, may be responsible for poor exercise capacity.

Almost 30% of CKD patients in the sample had T2DM; this was deemed ‘controlled’ by their acting clinician upon entry to the study, however, HbA_1c_ levels were not assessed. Given previous research investigating SMO_2_% in diabetic groups [9, 45, 50, 51], we conducted an exploratory analysis on patients with and without T2DM. Although diabetic patients had poorer exercise capacity, we observed no differences at baseline between the groups, nor any changes in SMO_2_% during exercise. However, recovery kinetics was markedly different with diabetic patients taking longer to recover. Indeed, overall, patients with T2DM never fully restored SMO_2_% to their baseline levels. This contrasts research by Mohler et al. [51] whom found no difference in recovery parameters between diabetic and non-diabetic patients; here differences were only seen in those with PAD, supporting previous work [23, 24]. The difference in recovery kinetics seen in our sample could be related to mitochondrial function. Reduced mitochondrial number and function are critically involved in the pathophysiology of diabetes [52], and given their role in oxidative phosphorylation, dysfunctional mitochondria may be responsible for the inadequate recovery seen following exercise. Dialysis-induced changes in muscle exercise performance have previously been proposed to depend exclusively on reduced mitochondrial oxidative capacity, rather than a defect in oxygen transport [45, 50].

Given the inability to distinguish between intercapillary Hb and intracellular Mb [20] contribution in our sample, our data reflects local tissue oxygenation inclusive of Hb and Mb. Whilst the relative contribution of Mb in the calf is considered minimal beyond the initial phase of exercise [23, 24], we consider our measurements to reflect a general view of SMO_2_%, which cannot address interactions between microvascular and intracellular O_2_ stores. It is also important to note that in the absence of absolute measures of muscle metabolism (i.e., ATP and PCr levels) we cannot distinguish between poor mitochondrial function and reduced muscle O_2_ conductance. Along with mitochondrial capacity, O_2_ delivery and muscle perfusion can also be modulated by the vasculature [10]. Endothelial dysfunction is well established in CKD groups [53] and consequently may contribute to poor exercise capacity by reducing the delivery of oxygenated Hb to the muscles [53]. Future studies should combine measures of mitochondrial capacity with endothelial function and regional blood flow measurements [9].

Limitations regarding SMO_2_% measurement and the effect of subcutaneous fat thickness have also been reported [9, 23, 24]. Adipose tissue metabolism is lower than muscle metabolism, which could lead to an inaccurate estimation of muscle O_2_ consumption by NIRS [54]. However, the typical penetration depth of NIRS signal generally can reach up to 25 mm depending on tissue composition [24], and research shows that even in morbidly obese patients, fat deposition in the lower leg region is low and an adipose tissue depth of > 20 mm is rare [54]. Whilst further supporting our use of the calf as a measuring site of SMO_2_%, this demonstrates NIRS has the ability to measure SMO_2_% despite extreme amounts of subcutaneous fat. Further research could utilize basic calf skin folds to help quantify subcutaneous fat mass. Whilst no patients in the current sample were anaemic, it is important to be aware that tissue O_2_ saturation and deoxygenation rate may be affected by anaemia. In anaemic patients, O_2_ delivery decreases and O_2_ extraction is increased. This leads to decreased venous Hb saturation and a lower tissue O_2_ saturation [55]. In healthy individuals, more pronounced muscle deoxygenation is observed during exercise in an acute hypoxic state [9]. No research exists in CKD populations, particularly exploring the role of anaemia on muscle tissue O_2_ kinetics during exercise.

As an exploratory sub-study, our sample is limited to only a small heterogeneous sample of patients with CKD recruited to the main trial [38]. Given the small range of eGFR in the sample, our data cannot explore the relationship between renal function and muscle O_2_ kinetics. To provide preliminary data between CKD and a ‘normal’ response, we recruited a small sample of HCs. To account for differences in age and sex distribution, we used these variables as covariant in our statistical analysis. It has been previously reported [56] that females have reduced muscle oxygen saturation due to smaller muscle mass, lower capillary density, lower Hb content, and lower oxidative potential than males. Given that our HC data (despite being made up largely of females) shows superior oxygen kinetics (including a slower desaturation and better recovery), our results may be underestimating the difference somewhat between the CKD and HC groups. Overall, our results show NIRS is an easily acceptable technique capable of inferring changes as a result of exercise in these patients. Further research could investigate patients with more severe disease progression. Although comparisons with a ‘healthy’ group was not a primary objective, we have shown there may be differences between patients with and without renal impairment. Future study should utilise an age- and sex-matched control group to investigate this further.

## Conclusion

We have shown, for the first time in CKD patients, that NIRS can be a low-cost-, simple, feasible, portable, and non-invasive means to measure skeletal muscle O_2_ kinetics during exercise. Variables such as the time taken to reach the minimum SMO_2_% value and recovery times may provide insights into dysfunctional O_2_ kinetics in CKD, including the mitochondrial capacity for oxidative phosphorylation. Future research should investigate the role of exercise training on these parameters, as well as investigate the role of NIRS-derived O_2_ kinetics and alternative in vivo mitochondria function. Whilst further investigation is needed into the reliability of the device, wireless NIRS appears to be a promising clinical tool can offer insights into key physiological and pathophysiological adaptations to conditions of increased or impaired muscle O_2_ kinetics.
